# Physiological Action of Spinal Accessory Demonstrated by Injury of Its Filaments

**Published:** 1882-12

**Authors:** W. S. Tremaine

**Affiliations:** U. S. A.


### Physiological Action of Spinal Accessory Demonstrated by Injury of its Filaments.

Reported by W. S. Tremaine, M. D., U. S. A.

W. G. T., a deputy United States marshal, was wounded in several places by a long, sharp pen-knife. A wound of the arm had become irritated by some application made to it by a quack. He then sent for me, and while examining his injuries I noticed he spoke in a hoarse whisper. He informed me that he lost his voice immediately after receiving his wounds. Closer examination revealed a small punctured wound on the right side of the neck, into which a probe could be introduced for about an inch. An injury to the pneumogastric at once suggested itself. Examination with the laryngoscope revealed the true state of the case: the right vocal cord was immovable on phonation, due, of course, to paralysis of the arytenoid, lateral, crico-arytenoid and thyro-arytenoid muscles. A constant current was applied by means of a laryngeal electrode daily for some weeks. Mr. T. was obliged, by his business, to leave my vicinity. I advised him to go to New York and consult my friend, Professor Clinton Wagner. This he promised to do when his business would permit, in the following autumn.

I saw nothing more of him for about four months, when he returned and informed me that he had fully recovered the use of his voice. I then examined him with the laryngoscope and found motion fully restored to the paralyzed muscles.

REMARKS.—It is not often that physiological functions are so accurately demonstrated in the living body, the point of interest being the localization of the injury to filaments of the spinal accessory.

In 1832 Bischoff demonstrated the influence of this nerve in phonation, afterwards confirmed by repeated and conclusive experiments of Bernard. These experiments were, of course, conducted on the lower animals.

The filaments of the spinal accessory reach the muscles of the larynx by means of the recurrent laryngeal; as section of the latter interferes with respiratory movements, and as there was, in the case under consideration, no disturbance of these, I have thought the case worth reporting, as demonstrating the physiological action of the spinal accessory in the human being. It must, of course, be evident that such a case is rare. I therefore had a photograph taken, showing the exact situation of this wound.

